# Molecular and morphological differentiation between two Miocene-divergent lineages of Amazonian shrimps, with the description of a new species (Decapoda, Palaemonidae, *Palaemon*)

**DOI:** 10.3897/zookeys.457.6771

**Published:** 2014-11-25

**Authors:** Fabrício Lopes Carvalho, Célio Magalhães, Fernando Luis Mantelatto

**Affiliations:** 1 Laboratory of Bioecology and Crustacean Systematics (LBSC), Postgraduate Program in Comparative Biology. Department of Biology, Faculty of Philosophy, Sciences and Letters at Ribeirão Preto (FFCLRP), University of São Paulo (USP), São Paulo, Brazil; 2 Instituto Nacional de Pesquisas da Amazônia, Coordenação de Biodiversidade, Manaus, Amazonas, Brazil

**Keywords:** Amazon basin, divergence time, freshwater shrimp, *Palaemon yuna*, taxonomy

## Abstract

*Palaemon carteri* (Gordon, 1935) and *Palaemon ivonicus* (Holthuis, 1950) are morphologically similar species of South American freshwater shrimps. Past studies have questioned the taxonomic status of both species, which are supposed to have partially sympatric geographic distributions in the Amazon basin. We analyzed a 550 bp fragment of the mitochondrial 16S rRNA gene from these Amazonian *Palaemon* species as well as from 11 palaemonids as the outgroup. Additionally, we checked diagnostic characters of the genus and family as well as other morphological characters that have been little explored before. *Palaemon carteri* and *Palaemon ivonicus* are allocated in two sister lineages, with wide genetic divergence and little morphological differentiation. The divergence time between these lineages was estimated as approximately 10 million years ago. Both molecular and morphological data support the taxonomic validity of both *Palaemon carteri* and *Palaemon ivonicus*, refuting the hypothesis of synonymy. In addition, a new species, *Palaemon yuna* **sp. n.**, closely related to *Palaemon ivonicus*, is described. Our findings indicate that these species can be differentiated using the projection of the anterolateral margin and anterolateral spine of the first antennular segment, shape of the rostrum, and relative size of the appendix masculina.

## Introduction

The genus *Palaemon* Weber, 1795 comprises 84 marine, estuarine and freshwater species in tropical and subtropical regions, including *Palaemonetes* Heller, 1869, which was recently considered to be a junior synonym of *Palaemon* by De Grave and Ashelby (2013), and the new species described here. Four strictly freshwater species occur in South America, distributed in the Amazon, Orinoco and Paraguay/lower Paraná River basins: *Palaemon carteri* (Gordon, 1935), *Palaemon ivonicus* (Holthuis, 1950) *Palaemon mercedae* (Pereira, 1986) and a new species described herein.

*Palaemon carteri* and *Palaemon ivonicus* are freshwater species with abbreviated larval development (see Magalhães 1986 for larval description of *Palaemon ivonicus*) and are supposed to occur sympatrically in the Amazon basin as well as being very similar morphologically. They have been distinguished primarily based on rostral characters and on the position of the branchiostegal tooth. Holthuis (1952) stated that *Palaemon ivonicus* has the lower margin of the rostrum with two or three teeth, the rostrum rather high and straight, and the branchiostegal tooth removed a considerable distance from the anterior margin of the carapace, with its tip failing to reach beyond this margin. On the other hand, *Palaemon carteri* has the lower margin of the rostrum with four to seven teeth; the rostrum slender, often curved upwards; and the branchiostegal tooth removed a short distance from the anterior margin of the carapace. Nevertheless, some studies have shown that the main characters currently used (position of the branchiostegal tooth, number of rostral teeth, and rostral shape) are not enough to differentiate *Palaemon ivonicus* from *Palaemon carteri* consistently (Gomes-Corrêa 1977, Odinetz-Collart and Enriconi 1993, García-Dávila and Magalhães 2003). Therefore, the high interspecific morphological similarity and intraspecific variability found in these species raised doubts as to whether these two nominal species represent distinct biological entities (Gomes-Corrêa 1977, Odinetz-Collart and Enriconi 1993, García-Dávila and Magalhães 2003, García-Dávila et al. 2005).

A multivariate morphometric approach was applied in order to confirm whether the two species constitute separate biological entities in the Amazon basin, and a wide plasticity and overlap in these characters among populations was found (García-Dávila et al. 2005), enhancing the hypothesis of synonymy (Gomes-Corrêa 1977). However, populations from black- and clear-water river systems (Negro and Tapajós river basins, respectively) formed a group slightly distinct from those from white-water river systems (Solimões/Amazon river basin), based on morphometric patterns (García-Dávila et al. 2005; see Sioli 1984 for a characterization of the three Amazonian hydrological systems).

The occurrence of *Palaemon ivonicus* in the Amazon, Orinoco and Paraguay/lower Paraná basins, however, might indicate a Miocene origin for these lineages. In this period the Orinoco and Amazon basin were widely connected and different sequences of capture of headwater might have resulted in dispersal of species across boundaries of the Amazon and Paraguay basins during the Tertiary (see Lundberg et al. 1998 and Magalhães et al. 2005), which means that these populations could have had enough time to undergo speciation.

Regarding the morphological variability of this group, our study aimed to test the taxonomic status of *Palaemon ivonicus* and *Palaemon carteri*, as well as to verify the presence of a new species from the Negro River basin, on the basis of partial sequences of the large ribosomal subunit 16S and morphological analyses.

## Methods

### Abbreviations and symbols used

CCDB Crustacean Collection of the Biology Department, Faculty of Philosophy, Sciences and Letters at Ribeirão Preto, University of São Paulo, Ribeirão Preto, Brazil.

CL Carapace length (measured from the posterior margin of the orbit to the posterior margin of the carapace).

CNCR National Crustacean Collection of the Instituto de Biología, Universidad Nacional Autónoma de México, Mexico City, Mexico.

INPA Instituto Nacional de Pesquisas da Amazônia, Manaus, Brazil.

OUMNH-ZC Zoological Collections of the Oxford University Museum of Natural History, Oxford, England.

MNRJ National Museum of the Federal University of Rio de Janeiro, Rio de Janeiro, Brazil.

MPEG Museu Paraense Emilio Goeldi, Belém, Brazil.

MV Museum of Victoria, Melbourne, Australia.

MZUSP Museum of Zoology of the University of São Paulo, São Paulo, Brazil.

NHM Natural History Museum, London, England.

USNM National Museum of Natural History (United States National Museum), Smithsonian Institution, Washington, D.C., USA.

MZUCR Universidad de Costa Rica, Museo de Zoología, San José, Costa Rica.

♂: male, ♀: female, ♀ov: ovigerous female.

### Sampling

**Figure 1.**
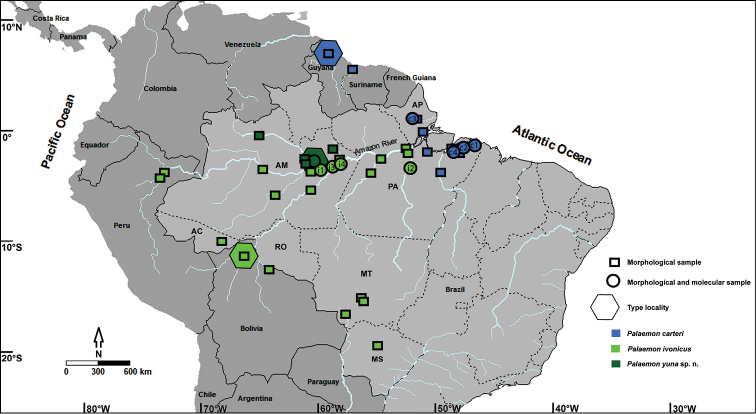
Sample sites of *Palaemon carteri*, *Palaemon ivonicus* and *Palaemon yuna* sp. n. c1–Bragança, Pará; c2–Santa Maria do Pará, Pará; c3–National Forest of Amapá, Amapá; c4–Belém, Pará; i1–Solimões River, near Manaus, Amazonas; i2–Xingu River, Altamira, Pará; i3 and i4–Itacoatiara, Amazonas; AC-Acre; AM-Amazonas; AP-Amapá; MS-Mato Grosso do Sul; MT-Mato Grosso; PA-Pará and RO-Rondônia.

Specimens from several localities were obtained from field collections as well as from visits to and loans from the above-mentioned carcinological collections (Fig. 1). Collected specimens were euthanized on ice, transferred to 96% ethanol, morphotyped under a Leica M205C stereomicroscope, and finally preserved in 80% ethanol for deposit in the CCDB. Specimens from donations and loans were also assigned to morphotypes, and each morphotype was deposited in the CCDB or in the original collection. The collections of species conducted in this study complied with current applicable state and federal laws of Brazil [FLC’s authorization from ICMBio (No. 25329); DIFAP/IBAMA/126/05; and permanent license to FLM for collection of Zoological Material No. 11777-1 MMA/IBAMA].

### Molecular data

DNA extraction, amplification and sequencing protocols followed Schubart et al. (2000) with modifications as in Mantelatto et al. (2007, 2009) and Pileggi and Mantelatto (2010). Total genomic DNA was extracted from the muscle tissue of the abdomen. An approximately 550 bp region of the mitochondrial 16S rRNA gene was amplified from four specimens of *Palaemon carteri*, four of *Palaemon ivonicus*, one of *Palaemon yuna* sp. n. and ten of other palaemonids (Table 1). The amplification was performed by polymerase chain reaction (PCR) in an Applied Biosystems Veriti 96 Well Thermal Cycler® (thermal cycles: initial denaturing for 5 min at 95 °C; pairing for 40 cycles: 45 s at 95 °C, 45 s at 52 °C, 1 min at 72 °C; final extension 5 min at 72 °C) with universal 16S mtDNA primers 1472 (5’-AGATAGAAACCAACCTGG-3’) (Crandall and Fitzpatrick 1996) and 16S-L2 (5’-TGCCTGTTTATCAAAAACAT-3’) (Schubart et al. 2002). PCR products were purified using Sure Clean (Bioline) and sequenced with the ABI Big Dye® Terminator Mix (Applied Biosystems, Carlsbad, CA) in an ABI 3730 XL DNA Analyzer (Applied Biosystems, Foster City, CA) following Applied Biosystems protocols. All sequences were confirmed by sequencing both strands. A consensus sequence for the two strands was obtained using the BIOEDIT software (version 7.0.5) (Hall 2005).

**Table 1.** Specimens of Palaemonidae used for the phylogenetic analyses. CCDB: Crustacean Collection of the Department of Biology of the Faculty of Philosophy, Sciences and Letters at Ribeirão Preto, University of São Paulo; CNCR: National Crustacean Collection of the Instituto de Biología, Universidad Nacional Autónoma de México; MPEG: Museu Paraense Emilio Goeldi; MV: Museum Victoria; MZUCR: Museo de Zoología, Universidad de Costa Rica.

Taxon	Locality	Collection accession number	GenBank
*Creaseria morleyi* (Creaser, 1936)	Yucatan Peninsula, Mexico	---	EU448997
*Palaemon carteri* (Gordon, 1935)	(c1) Jequiri, Bragança, Pará, Brazil	MPEG 0787	KF923721
*Palaemon carteri* (Gordon, 1935)	(c2) Santa Maria do Pará, Pará, Brazil	CCDB 4339	KF923720
*Palaemon carteri* (Gordon, 1935)	(c3) Japim stream, National Forest of Amapá, Amapá, Brazil	MPEG 1108	KF923727
*Palaemon carteri* (Gordon, 1935)	(c4) Mocambo, Belém, Pará, Brazil	MPEG 0628	KF923730
*Palaemon gracilis* (Smith, 1871)	Pacific coast, Costa Rica	CCDB 3402	KF923714
*Palaemon hancocki* Holthuis, 1950	Golfo Dulce, Puntarenas, Pacific coast, Costa Rica	MZUCR 2477-02	KF923715
*Palaemon intermedius* (Stimpson, 1860)	Victoria, Australia	MV J60843	KF923725
*Palaemon ivonicus* (Holthuis, 1950)	(i1) Solimões River, Manaus, Amazonas, Brazil	CCDB 1435	KF923717
*Palaemon ivonicus* (Holthuis, 1950)	(i2) Xingu River, Altamira, Pará, Brazil	MPEG 0715	KF923726
*Palaemon ivonicus* (Holthuis, 1950)	(i3) Poranga, Itacoatiara, Amazonas, Brazil	CCDB 4632	KF923728
*Palaemon ivonicus* (Holthuis, 1950)	(i4) Itacoatiara, Amazonas, Brazil	CCDB 4716	KF923729
*Palaemon kadiakensis* (Rathbun, 1902)	Convent, Louisiana, USA	CCDB 1600	KF923718
*Palaemon longirostris* H. Milne Edwards, 1837	Guadiana River, Portugal	CCDB 2750	KF923724
*Palaemon northropi* (Rankin, 1898)	Mamanguape River, Paraíba, Brazil	CCDB 4332	KF923722
*Palaemon pandaliformis* (Stimpson, 1871)	Ilha Comprida, São Paulo, Brazil	CCDB 813	KF923713
*Palaemon pugio* (Holthuis, 1949)	River Delta, Gautier, Mississippi, USA	CCDB 3804	KF923723
*Palaemon ritteri* Holmes, 1895	Bahía Wafer, Puntarenas, Pacific coast, Costa Rica	MZUCR 2396-04	KF923719
*Palaemon suttkusi* (Smalley, 1964)	Salado River, Zaragoza, Mexico	CNCR 25864	KF923712
*Palaemon yuna* sp. n.	Lago Tupé beach, Negro River, Manaus, Amazonas, Brazil	CCDB 4866	KF923716

### Phylogenetic analysis

Sequence alignments were conducted in MAFFT alignment software (version 7.058) with default settings (Katoh and Standley 2013). Estimates of uncorrected genetic divergence (p-distance) for sequence pairs were conducted in MEGA (version 5.2.2) (Tamura et al. 2011), and statistical selection of models of nucleotide substitution in jModelTest (version 2.1.4) (Darriba et al. 2012). The gene dataset was tested for nucleotide substitution saturation using the test by Xia et al. (2003) implemented in DAMBE v. 5.3.48, which revealed no significant saturation (P < 0.05) for symmetrical trees.

We performed four phylogenetic reconstructions: two by Bayesian inference, one by maximum likelihood and one by parsimony. All analyses were conducted using *Creaseria morleyi* (Creaser, 1936) as the outgroup. A consensus tree of the two Bayesian and maximum likelihood analyses was constructed by 50% majority-rule in the Mesquite package (2.75, build 566). Only posterior probabilities and bootstrap values above 50% are shown. All other software settings not mentioned below were maintained as default.

The Bayesian inferences were performed in the MrBayes software (version 3.2.2) (Ronquist et al. 2012). The Metropolis-coupled Markov chain Monte Carlo was used to empirically determine the posterior probability distribution of trees, branch lengths and substitution parameters. The nucleotide substitution model assumed was the 4by4 with general time-reversibility, gamma-distributed rate variation across sites and invariable sites (GTR+Γ+I). Five gamma rate categories were used. The prior probability distributions on the parameters of the model were maintained as default. Aiming to evaluate possible effects of overfitting, we also performed a second Bayesian inference using a simpler model (Hasegawa-Kishino-Yano with gamma-distributed rates: HKY+Γ) indicated by the Bayesian information criterion. In this second analysis, the parameter values given by jModelTest (nucleotide frequencies, transition/transversion ratio, shape of the gamma distribution and proportion of variable sites) were used to define the prior probability distributions on the parameters of the model. Both analyses were carried out with 10^7^ generations in two independent runs, with one cold and four heated parallel chains. The parameter values were saved once every 1,000 rounds. The runs were stopped if they had reached stationarity (average standard deviation of the split frequencies below 0.01). The first quarter of parameters and trees was discarded (burn-in of 25%); see Ronquist et al. (2009) for further details.

The maximum likelihood (ML) analysis was conducted in the RAxML program (7.6.3) (Stamatakis 2006) implemented in CIPRES (“Cyberinfrastructure for Phylogenetic Research”; http://www.phylo.org). The consistency of topologies was measured by the bootstrap method. The number of bootstrap replicates (650) as well as the proportion of invariable sites was defined by RAxML.

The maximum parsimony analysis was performed using the branch-and-bound algorithm in PAUP (version 4.0 for Unix/Linux). We conducted a bootstrap analysis with 1,000 replicates. Gaps, missing and ambiguous characters were excluded in this analysis.

## Molecular dating

The likelihood ratio test (LRT) was used to test the null hypothesis that the data evolved under a molecular clock. The likelihoods for the molecular trees, under the GTR+Γ+I model, with and without an imposed molecular clock were compared using the LRT calculator in jModelTest. The assumption of a molecular clock could not be rejected (P = 0.51). We used both a strict molecular clock and a relaxed molecular clock with independent gamma rates (IGR) in MrBayes, under the settings mentioned above, in order to estimate an approximate divergence time between the *Palaemon carteri* and *Palaemon ivonicus* / *Palaemon yuna* lineages. We assumed the rate of 0.0083 substitutions per site per million years to corrected divergence values for the 16S rRNA gene. This rate was estimated for neotropical palaemonid species of *Palaemon*, assuming that the isolation of the transisthmian estuarine sibling species *Palaemon ritteri* Holmes, 1895 and *Palaemon paivai* Fausto Filho, 1967 (CCDB 4334) occurred about three million years ago (Carvalho et al. unpublished data), as demonstrated for other estuarine carideans (Knowlton and Weigt 1998, Hurt et al. 2009). This rate is also compatible with rates estimated for other decapods (0.006–0.009) by Schubart et al. (2000) for the same gene.

### Morphological analysis

Once the phylogenetic relationships based on molecular data were known, we analyzed adult morphological characters of South American species of *Palaemon* in order to verify the morphological support for the clades obtained, as well as provide new diagnoses for the *Palaemon carteri* / *Palaemon ivonicus* / *Palaemon yuna* group.

We checked out the diagnostic characters that were traditionally used to differentiate *Palaemon carteri* from *Palaemon ivonicus*: position of the branchiostegal tooth, number of rostral teeth, and rostral shape (Holthuis 1952, Melo 2003). We also analyzed other characters that were traditionally used in Palaemonidae, as well as others that have been little explored in the taxonomy of the group, such as the projection of the anterolateral margin of the first antennular segment, the anterolateral spine of the first antennular segment, and the relative size of the appendix masculina.

The search for morphological differences among species was conducted using the optimized comparison method described below. Initially, all pairwise differences found between the specimens from each clade in the tree were listed. Those differences with more than 80% constancy were chosen for further validation, using the remaining specimens from the same lots used in the molecular analyses. The consistency of each difference was evaluated step-by-step, analyzing blocks of 10 specimens from each clade. Characters with less than 80% constancy were discarded and no longer analyzed in the following blocks. Additional blocks of specimens from lots that were not included in the molecular analyses were used for a final check.

The diagnostic characters found were verified in the original descriptions (Gordon 1935, Holthuis 1950a, 1952) as well as in the type series of *Palaemon carteri* and *Palaemon ivonicus* in order to confirm the taxonomic entity of each clade.

## Results

The two Bayesian analyses, maximum likelihood and parsimony analyses indicated the same topology. The mean standard deviation of the split frequencies after 10^7^ generations was less than 0.003 for both GTR+Γ+I and HKY+Γ models. No important differences were found between the two Bayesian inferences using each model, since only slight differences were noted in the posterior probabilities of the clades and branch lengths, indicating that there is no perceptible effect of model overfitting. Similarly, only dissimilarities in the values of node support were observed between the maximum likelihood and the Bayesian inferences, with the GTR+Γ+I implemented in both analyses.

Of the 435 aligned positions (after exclusion of gaps, missing and ambiguous characters), 193 sites were variable, of which 54 (28%) were parsimony-informative. The parsimony reconstruction also showed more than 50% bootstrap values for all clades indicated by the previous analyses, with high support for most of them.

**Figure 2.**
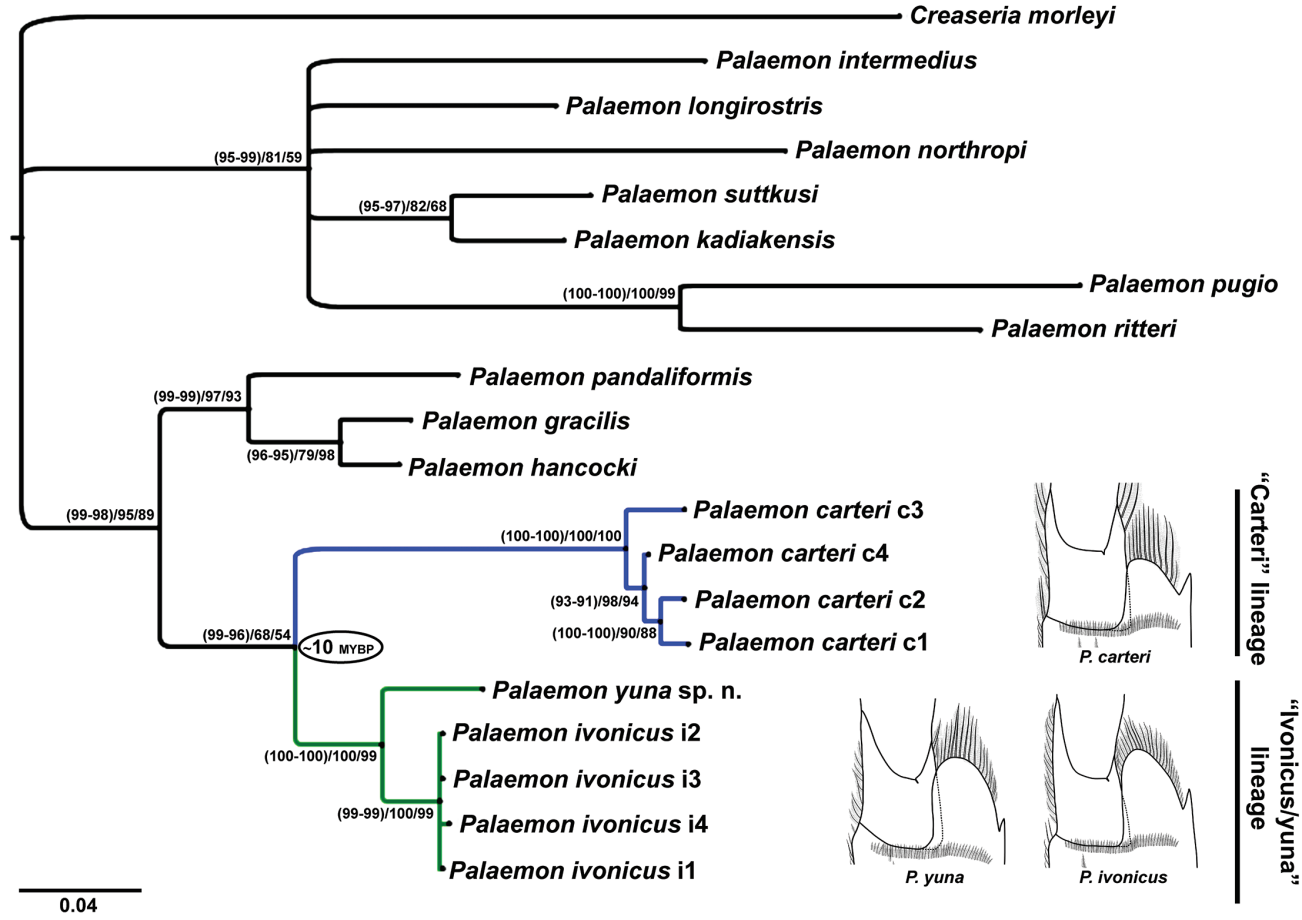
Bayesian (GTR+Γ+I and HKY+Γ models) and maximum likelihood 50% majority-rule consensus tree. Numbers in the nodes represent posterior probabilities (GTR+Γ+I and HKY+Γ, respectively), and bootstrap value for maximum likelihood and parsimony analyses, respectively. c1–Bragança, Pará; c2–Santa Maria do Pará, Pará; c3–National Forest of Amapá, Amapá; c4–Belém, Pará; i1–Solimões River, near Manaus, Amazonas; i2–Xingu River, Altamira, Pará; i3 and i4–Itacoatiara, Amazonas. MYBP–million years before present.

**Figure 3.**
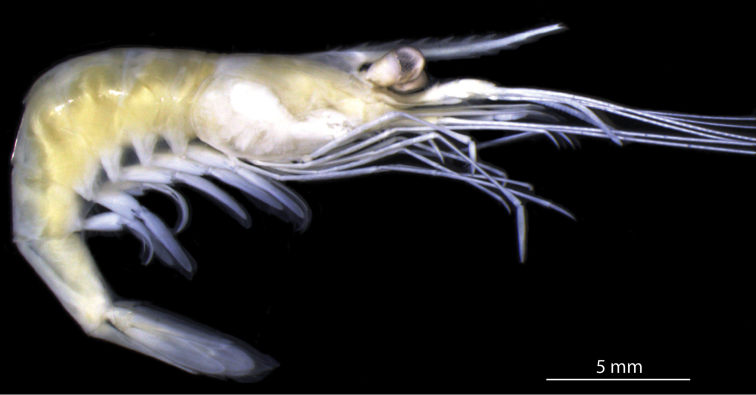
*Palaemon yuna* sp. n. Holotype, male, CCDB 4865, habitus, lateral view.

The monophyly of the *Palaemon carteri* / *Palaemon ivonicus* / *Palaemon yuna* group was indicated in all analyses, although this clade had weak bootstrap support in the parsimony and maximum-likelihood methods. All analyses supported the existence of two sister lineages for the specimens of this group (Fig. 2). The “carteri” lineage allocated specimens from eastern Amazonia, assigned to *Palaemon carteri*. The “ivonicus/yuna” lineage comprised specimens from central and western Amazonia, which were assigned to *Palaemon ivonicus* and *Palaemon yuna* sp. n. The population from the black water Negro River, assigned to *Palaemon yuna* sp. n. (Fig. 3), clearly fell outside the clade that comprised specimens of *Palaemon ivonicus* from other regions of the Amazon basins (with white and clear water).

**Figure 4.**
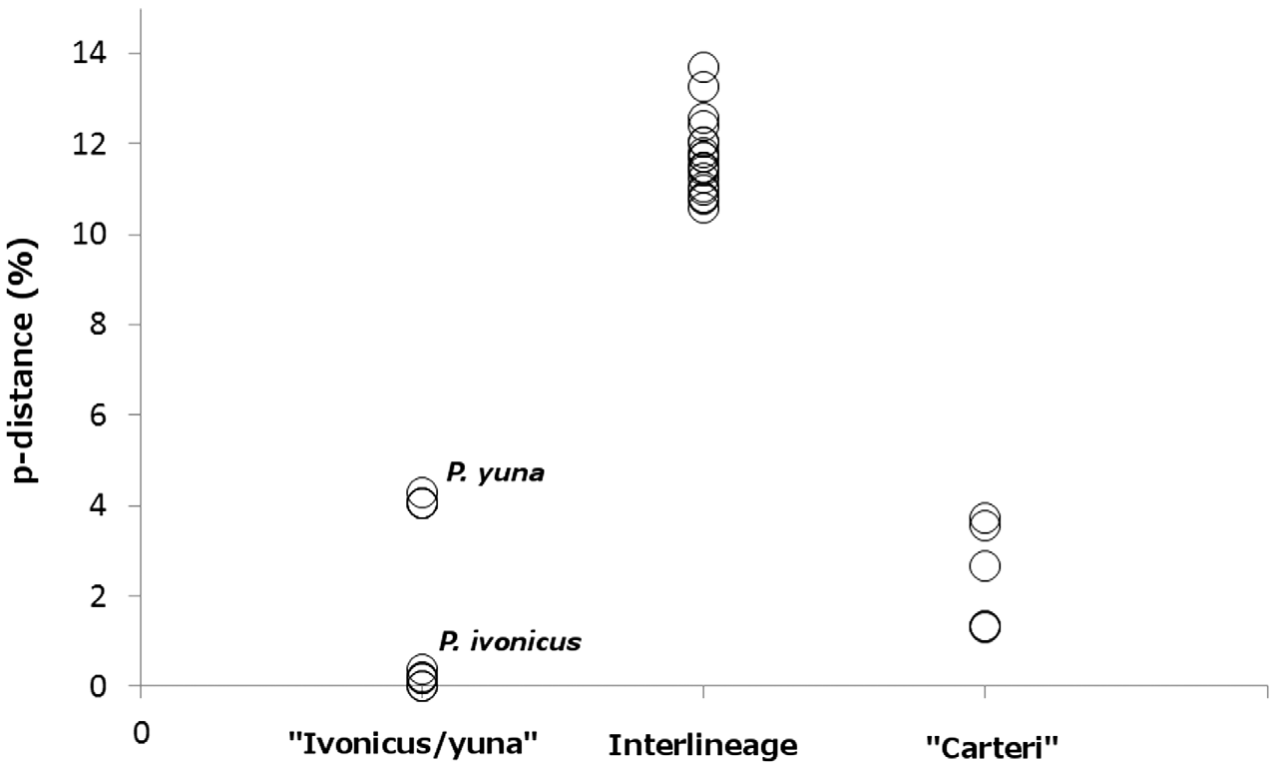
Intralineage and interlineage uncorrected genetic distance values for the “ivonicus/yuna” and “carteri” lineages.

The wide genetic dissimilarity between the “carteri” and “ivonicus/yuna” lineages (from 10.6% to 13.7%), compared among the representative members of the genus used here (from 3.5% to 23.0%; data not shown), also supports the hypothesis that *Palaemon carteri* and *Palaemon ivonicus* are valid species, as well as clearly genetically divergent (Fig. 4). The intralineage genetic variability was 0–3.6% in “carteri” and 0–4.3% in “ivonicus/yuna”. The interspecific dissimilarity between *Palaemon ivonicus* and *Palaemon yuna* was 4.1–4.3%.

The divergence time between the “carteri” and “ivonicus/yuna” lineages, based on the 16S rRNA gene, was estimated as approximately 10 million years ago. This mean value varied between ~9 and ~11 Ma in our analysis, depending on the molecular-clock model used, as well as other parameter settings such as node constraints. The 95% credible intervals (highest posterior density–HDP) were 5.5–14.3 Ma using a relaxed clock, and 8.2–14.0 Ma using a strict clock.

**Figure 5.**
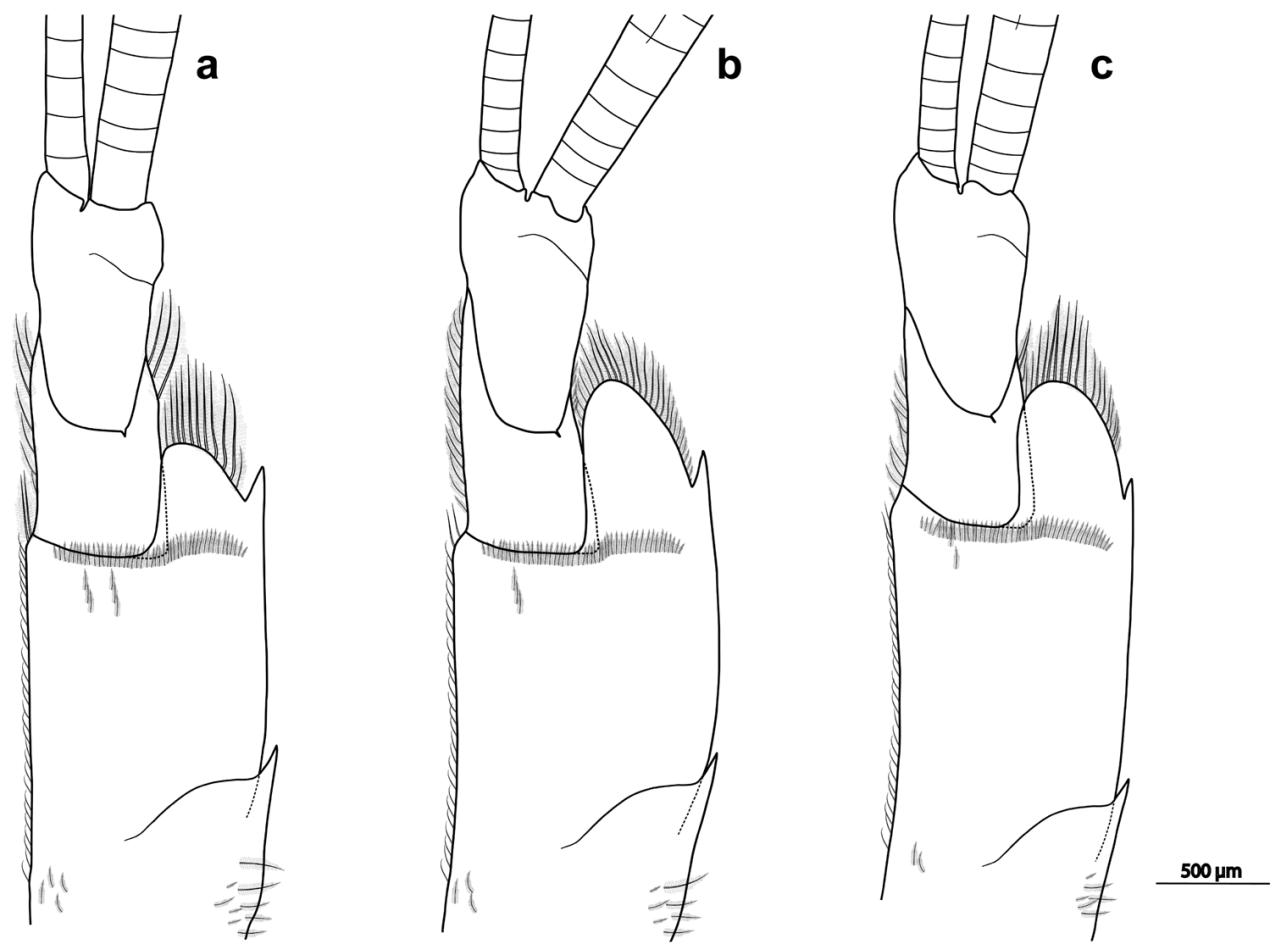
Commonest shape of the antennular peduncle. *Palaemon carteri* (**a** MPEG 787), *Palaemon ivonicus* (**b** INPA 128) and *Palaemon yuna* sp. n. (**c** CCDB 4866).

Morphological characters supported these lineages. The morphological analysis (including the type series of all three species) of 122 specimens of *Palaemon carteri*, 333 specimens of *Palaemon ivonicus* and 125 specimens of *Palaemon yuna* sp. n. indicated that the projection of the anterolateral margin and the anterolateral spine of the first antennular segment were useful characters to differentiate these lineages, as long as adult specimens were considered. The “carteri” lineage had the projection of the anterolateral margin of the first antennular segment slightly shorter, not reaching the dorsal distal margin of the second segment. Additionally, the anterolateral spine of the first antennular segment usually reached the middle of the projection of the anterolateral margin (Fig. 5a). On the other hand, the “ivonicus/yuna” lineage had the projection of the anterolateral margin of the first antennular segment longer, reaching the dorsal distal margin of the second segment. Moreover, the anterolateral spine of the first antennular segment did not reach the middle of the projection of the anterolateral margin (Fig. 5b–c).

**Figure 6.**
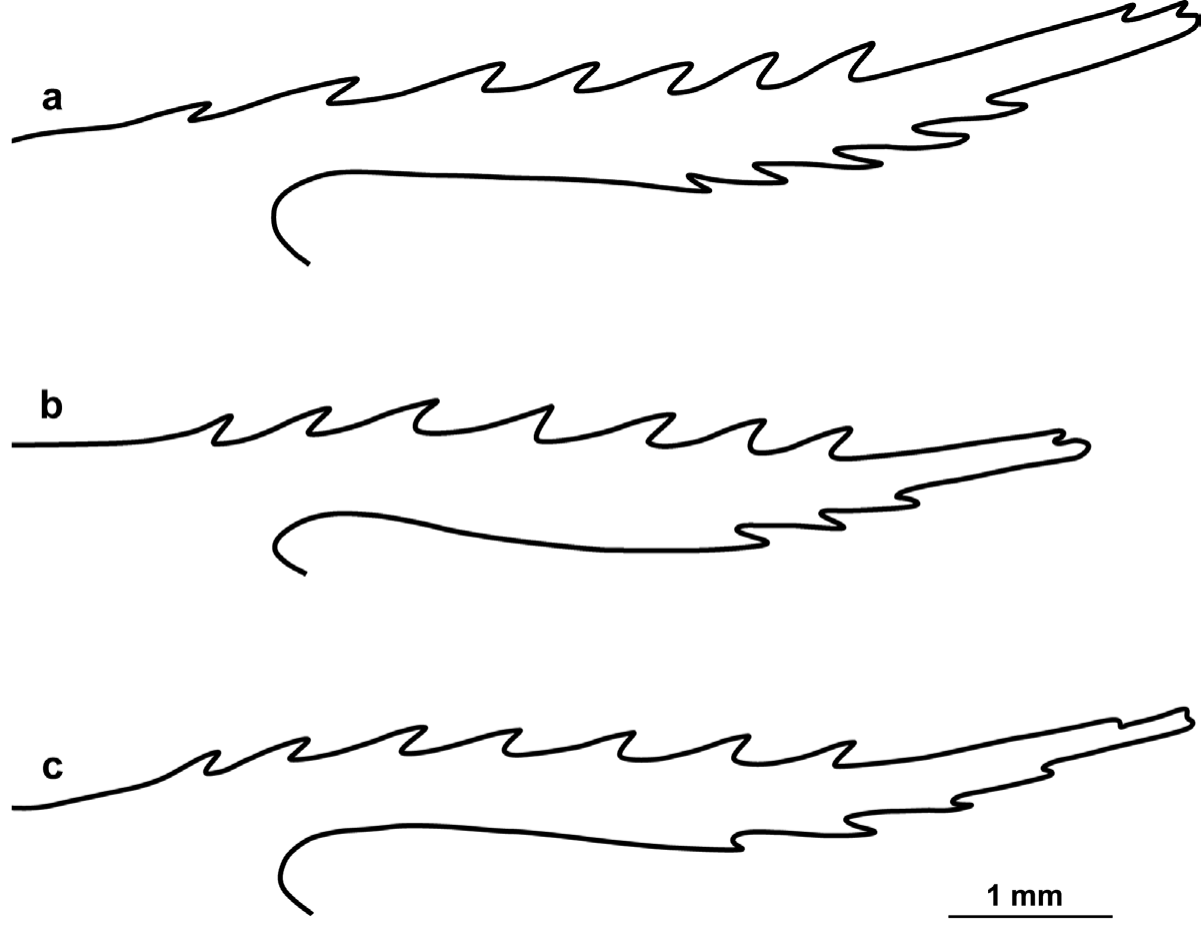
Commonest shape of the rostrum. *Palaemon carteri* (**a** MPEG 787), *Palaemon ivonicus* (**b** INPA 128) and *Palaemon yuna* sp. n. (**c** CCDB 4866).

The rostral characters (shape, relative size and number of ventral teeth of the rostrum) were helpful to differentiate *Palaemon ivonicus* from *Palaemon yuna* sp. n. (Fig. 6). *Palaemon ivonicus* had the rostrum high, straight or slightly curved upward, not overreaching the scaphocerite; the ventral margin had one to four teeth, usually three or fewer. On the other hand, *Palaemon yuna* sp. n. had the rostrum slightly curved upward, overreaching the scaphocerite; the ventral margin has two to five teeth, usually three or four. The relative length of the appendix masculina was also useful to distinguish adult individuals of the “ivonicus/yuna” lineage, since the appendix masculina reached up to 1.5 times the length of the appendix interna in *Palaemon ivonicus* and up to 1.1 times in *Palaemon yuna* sp. n.

Once the validity of *Palaemon ivonicus* and the new species for the Negro River basin were corroborated, we provide illustrations, diagnoses, identification key as well as a description of *Palaemon yuna* sp. n. in order to differentiate the three species from each other as well as from other South American species of *Palaemon*.

### Palaemon carteri

Taxon classificationAnimaliaDecapodaPalaemonidae

(Gordon, 1935)

Figures 5a and 6a

Palaemonetes carteri Gordon, 1935: 324, fig. 12;–Holthuis 1948: 113;–Holthuis 1950b: 32;–Holthuis 1966: 6 [part, spec. from Rio Tapajós];–Rodríguez 1980: 126;–Rodríguez 1981: 47 [in list];–Rodríguez 1982: 390;–Coelho and Ramos-Porto 1985: 408 [in table];–Holthuis 1993: 8;–Delgado et al. 1997: 16 [in list];–Ramos-Porto and Coelho 1998: 337 [in list];–Barros and Pimentel 2001: 20 [in list];–Vieira 2003: 61;–Magalhães and Pereira 2007: 9, 10, 12 [in list]; Mora-Day et al. 2009: 196 [in list];–Pereira et al. 2010a: 606 [in list];–Pereira et al. 2010b: 84 [in list];–Pileggi et al. 2013: 569 [part, material from Amapá and Bragança; ? material from rio Tapajós basin].
Palaemonetes (Palaemonetes) carteri –Holthuis 1950c: 10 [in list];–Holthuis 1952: 218, pl. 52, figs c-o, pl. 53, figs a–c;–Holthuis 1959: 81, text-fig. 9;–Kensley and Walker 1982: 11 [part, ? spec. from Rio Curua Una];–López and Pereira 1996: 54, fig. 8;–López and Pereira 1998: 77 [in list]; Melo 2003: 382 [part, not Amazonas].

#### Holotype.

Guyana, upper Cuyuni River, ♂, col. GS Carter (NHM 1935.5.20.19).

#### Paratypes.

Karow Creek, 2 m NE of Penal Settlement, Mazaruni, 1 ♂; River Cuyuni, 1♀ov; Forest Swamp, upper Cuyuni, 1♂; same data as holotype, 7♂ 3♀ 1♀ov 1 juvenile (NHM 1935.5.20.20-29).

#### Other material.

***Suriname*.** *Nickerie*. Lower Naui Kreek, Southern Niew, 10♂ 10♀, col. DC Geijskes, 18 March 1971 (INPA 176). ***Brazil*.** *Amapá*. Floresta Nacional do Amapá, igarapé Japim, 5♂ 5♀, col. CRM Santos and JEM Nanzelor, 27 October 2009 (MPEG 1108); Porto Grande, Floresta Nacional do Amapá, tributary of rio Araguari, 1♂ 4♀, col. CRM Santos, 28 October 2009 (CCDB 2755); Macapá, stream in the home of Sr. Marcondes, 1♂ 4♀ov, col. J Cunha, 6 March 2005 (MZUSP 17676). *Pará*. Barcarena, Vila do Conde, 5♂ 2♀ 3♀ov, col. B Mascarenhas, 23 March 2002 (MPEG 739); Belém, Mocambo, 5♂ 5♀ov, col. FR Pimentel and R Maia, January 9 1998 (MPEG 528); Belém, Mocambo, Reserva Mocambo, 4♂ 6♀, col. FR Pimentel and J Dias, 18 June 1999 (MPEG 628); Bragança, Jequeri, Sítio Anacuã, 7♂ 9♀, 23 October 2002 (MPEG 787); Ilha do Marajó, cachoeira do Arari, 1♀, col. J Cunha and J Zuanon, 16 May 2008 (MZUSP 22753); Castanhal, 1♀ov, col. FL Carvalho et al., 14 December 2012 (CCDB 4338); Ilha de Marajó, cachoeira do Arari, rio Arari, igarapé Popudas, 1♂ 1♀, col. J Cunha and J Zuanon, 17 May 2006 (MZUSP 23224); Laranjal do Jari, igarapé Arapiranga, 2♀ 1♀ov, col. Moreira et al., 25 March 2008 (MZUSP 23225); Melgaço, Floresta Nacional de Caxiuanã, 5♂ 4♀, 8 November 1999 (MPEG 717); Santa Maria do Pará, 3 juveniles, col. FL Carvalho et al., 15 December 2012 (CCDB 4339); Tucuruí, rio Tocantins basin, igarapé Santos, 2♀, col. W Zuink and LCF Alvarenga, 16 September 1984 (MNRJ 23382).

#### Diagnosis.

Mandibular palp absent. Rostrum slender, curved upward, reaching or just overreaching the tip of the scaphocerite; dorsal margin with 6 to 10 teeth; ventral margin with 3 to 7 teeth, usually 4 or more. Projection of the anterolateral margin of the first antennular segment overreaching the middle of the second segment, but not reaching, sometimes almost reaching, the dorsal distal margin of the second segment; anterolateral spine of the first antennular segment usually reaches the middle of the projection of the anterolateral margin. Appendix masculina up to 1.1 the length of the appendix interna, measured from their junction. Telson carrying 2 plumose setae between the inner distal stout setae; inner distal stout setae overreaching the distal tip of the telson.

#### Geographic distribution.

Venezuela (Amazonas, Bolívar, Delta Amacuro, Monagas), Guyana, Suriname, French Guiana, Brazil (eastern Amazon: Amapá, Pará).

#### Ecological features.

Usually associated with riparian vegetation, leaf litter and similar microhabitats in lakes, streams and rivers, in areas with low flow. At least in the Amazon river basin, its occurrence is usually associated to clear water river systems.

### Palaemon ivonicus

Taxon classificationAnimaliaDecapodaPalaemonidae

(Holthuis, 1950)

Figures 5b and 6b

Palaemonetes ivonicus Holthuis, 1950a: 98;–Holthuis 1966: 4, fig. 1;–Rodríguez 1981: 47 [in list];–Coelho and Ramos-Porto 1985: 408 [in table];–Ramos-Porto and Coelho 1990: 99 [part, not Rio Negro, Catagalo];–Kochalka et al. 1996: 113 [in list];–Ramos-Porto and Coelho 1998: 337 [in list]; Magalhães 1999: 36, 85 [in list];–Magalhães 2000: 59 [in list];–Magalhães 2001: 70, 133 [in list];–Magalhães 2002: 1096, figs 5, 6;–García-Dávila and Magalhães 2003: 675, figs 21–27, 55;–Magalhães and Pereira 2007: 9, 10, 12 [in list];–Valencia and Campos 2010: 224, figs 3–4;–Pileggi et al. 2013: 570.
Palaemonetes carteri –Holthuis 1966: 6 [part, ? 2 spec. from Lago Redondo];–Magalhães 2005: 69, 71 [in list]; Pileggi et al. 2013: 569 [? part, material from rio Xingu and rio Trombetas].
Palaemonetes (Palaemonetes) ivonicus –Holthuis 1950c: 10 [in list];–Holthuis 1952: 222, pl. 53, figs d–h;–Melo 2003: 382.
Palaemonetes (Palaemonetes) carteri –Kensley and Walker 1982: 11 [part., ? spec. from Rio Madeira].

#### Holotype.

Bolivia, Beni, Ivon, Beni River, ♀ (CL 7.5 mm), col. WM Mann, February 1922 (USNM 85234).

#### Paratype.

same data as holotype, 1♀ (CL 6.6 mm) (USNM 85234).

#### Other material.

***Brazil*.** *Acre*. Bujari, igarapé Mapinguari, 2♂ 18♀, col. LR Malabarba et al., 8 August 2001 (UFRGS 3179). *Amazonas*. Itacoatiara, canal Irandiba, 3♂, col. GY Hattori, April 2008 (CCDB 2753); Itacoatiara, igarapé Aeroporto, 2♂ 18♀ (CCDB 4725); Itacoatiara, Poranga, 7♂ 23♀ (CCDB 4632); Itacoatiara, 3♂ 15♀ (CCDB 4716); rio Madeira, Borba, 1♂ 9♀ (MNRJ 1078); rio Solimões, igarapé do Xiboreno, 1♂ 1♀, col. FL Carvalho and EA Souza-Carvalho, 28 January 2012 (CCDB 1435); rio Solimões, lago Janauacá, 1♀, col. J Donnath, 18 March 1978 (MZUSP 8183); rio Solimões, lago do Jacaré, 2♀, col. H Reichardt, 29 March 1967 (MZUSP 6405); Tefé, igarapé da Aeronáutica, 12♂ 8♀, col. JO Chaves, 21 March 1979 (INPA 128). *Pará*. Almeirim, rio Arraiolos, pesqueiro São Paulo, 7♂ 5♀, col. J Carvalho Júnior, 26 July 1999 (MPEG 689); Altamira, rio Xingu, 4♀, col. RM Sousa and Dionísio, 18 December 2000 (MPEG 715); Porto de Moz, rio Xingu, 1♂ 1♀ov, col. R Robles et al., 25 September 2013 (CCDB 4867); rio Tapajós, near the rio Cupari’s mouth, downstream Itaiatuba, 1♀, col. C Magalhães and LH Py-Daniel, 27 October 1991 (INPA 1176); Santarém, igarapé do Juá, 2♀, col. LM Sousa and JL Birindelli, 13 November 2006 (MZUSP 28358). *Rondônia*. Rio Guaporé, 1♂ 2♀, col. JC Malta, 25 September 1985 (INPA 326). *Mato Grosso*. Acorizal, 17♂ 6♀, col. Sebastiana, 28 August 1987 (MNRJ 1151); Acorizal, 9♂ 5♀ 4 juveniles, col. Sebastiana (MNRJ 1153); baía do Pio, Pantanal, 5♂ 15 juveniles, col. Sebastiana (MNRJ 1152); Poconé, baía do Pio, 14♂ 33♀ 19 juveniles (INPA 328). *Mato Grosso do Sul*. Rio Negro, córrego Anhumas, 9♂ 11♀, col. C Magalhães et al., 28 August 1998 (CCDB 4667). ***Peru*.** *Loreto*. Lago Urcococha, rio Amazonas, 10♂ 5♀, col. C García-Dávila, 10 January 1999 (INPA 883); Quistococha, río Itaya, 12♂ 7♀ov, col. C García-Dávila, 13 July 1998 (INPA 882).

#### Diagnosis.

Mandibular palp absent. Rostrum high, straight or slightly curved upward, not overreaching the scaphocerite; dorsal margin with 6 to 10 teeth; ventral margin with 1 to 4 teeth, usually 3 or fewer. Projection of the anterolateral margin of the first antennular segment reaching or overreaching the dorsal distal margin of the second segment; anterolateral spine of the first antennular segment almost reaching or overreaching the first third of the projection of the anterolateral margin. Appendix masculina up to 1.5 the length of the appendix interna, measured from their junction. Telson carrying 2 plumose setae between the inner distal stout setae; inner distal stout setae overreaching the distal tip of the telson.

#### Geographic distribution.

Venezuela? (Delta Amacuro, Monagas), Colombia? (Amazonas, Arauca, Casanare, Guainía, Meta, Vichada), Brazil (Acre, Amazonas, Mato Grosso, Mato Grosso do Sul, Pará), Bolívia (Beni, Cochabamba, Pando), Peru (Loreto, Madre de Díos), Paraguay.

#### Ecological features.

Usually associated with riparian vegetation, leaf litter and similar microhabitats in lakes, streams and rivers with white or clear water, in areas with low flow. In the western portion of the Amazon river basin it is commonly found in the floodplains of the white water river systems.

### Palaemon yuna sp. n.

Taxon classificationAnimaliaDecapodaPalaemonidae

http://zoobank.org/1541C001-E4DD-4812-9D60-277706CE391D

**Figure 7.**
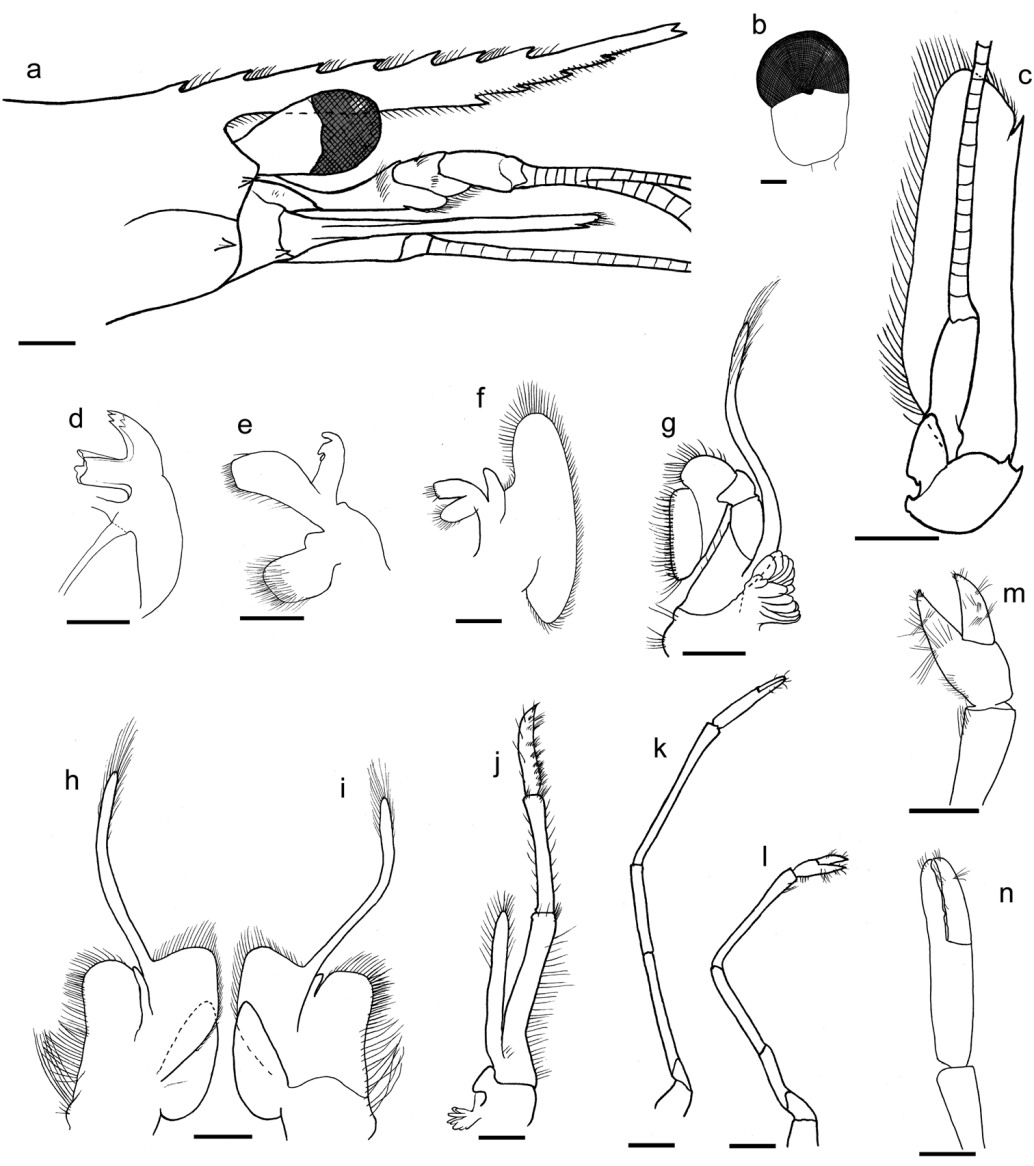
*Palaemon yuna* sp. n. Figure **a** holotype; figures **b–n** paratype (CCDB 4866, male, CL 5.5 mm). **a** anterior part of the carapace **b** right eye, dorsal view **c** left scaphocerite, ventral view **d** left mandible, ventral view **e** left maxillula, ventral view **f** left maxilla, ventral view **g** left second maxilliped, ventral view **h** left first maxilliped, ventral view **i** left first maxilliped, dorsal view **j** right third maxilliped, ventro-lateral view **k** right second pereiopod, ventro-lateral view **l** right first pereiopod, ventro-lateral view **m** right first chela, mesial view **n** right second chela, mesial view. Scale bar: **a, c, k** equal to 1 mm; others equal to 0.5 mm.

**Figure 8.**
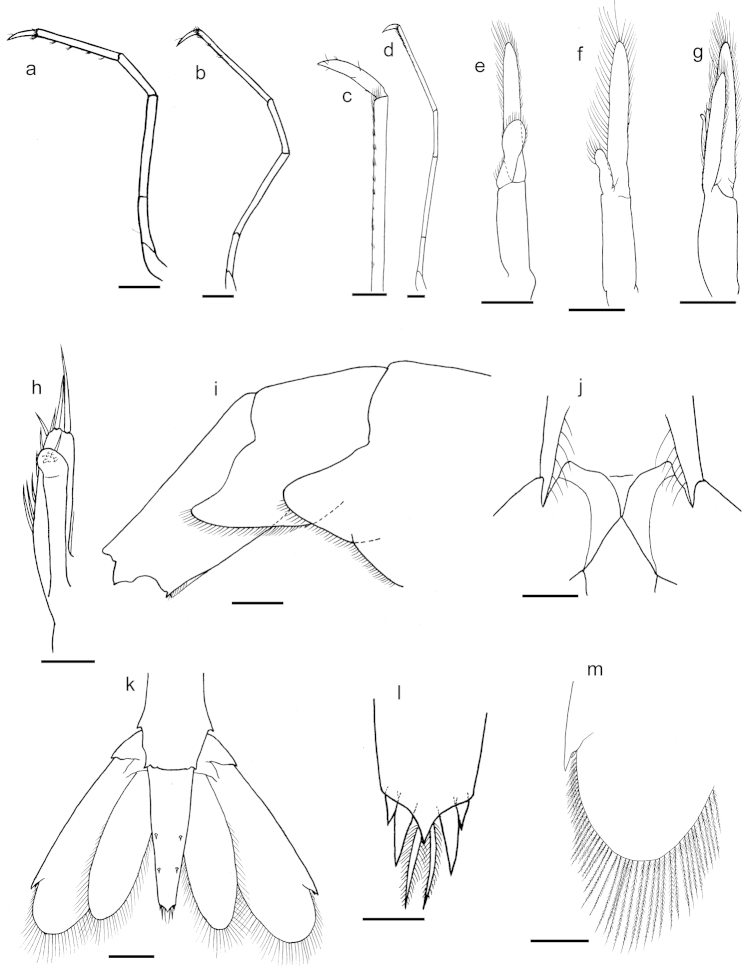
*Palaemon yuna* sp. n. Figures **i** and **m** holotype; figures **a–e, g, h, j–l** paratype (CCDB 4866, male, CL 5.5 mm); figure **f** paratype (CCDB 4866, female, CL 5.5 mm). **a** left third pereiopod, lateral view **b** left fourth pereiopod, lateral view **c** distal portion of the left fifth pereiopod, lateral view **d** left fifth pereiopod, lateral view **e** left first pleopod, posterior view **f** left first pleopod, posterior view **g** left second pleopod, posterior view **h** left appendix masculina and appendix interna, posterior view **i** right posterior part of the abdomen, lateral view **j** pre-anal fig, ventral view **k** telson and uropods, dorsal view **l** distal part of the telson, dorsal view **m** left distal portion of the exopod of the uropod, dorsal view. Scale bar: **a, b, d–g, i, k** equal to 1 mm; **c, j, m** equal to 0.5 mm; **h, l** equal to 0.25 mm.

Figures 3 , 5c , 6c , 7 and 8

Palaemonetes (Palaemonetes) carteri –Kensley and Walker 1982: 11, figs 13–14 [part, at least spec. from Rio Negro and its basin]; Melo 2003: 382 [part, Amazonas].
Palaemonetes carteri –Ramos-Porto and Coelho 1990: 99 [Rio Negro, Cantagalo; igarapé afluente do Rio Mapiri, Santarém?];–Pileggi et al. 2013: 569 [? part, material from rio Tapajós basin, rio Xingu and rio Trombetas].
Palaemonetes ivonicus –Ramos-Porto and Coelho 1990: 99 [part, Rio Negro, Cantagalo].

#### Holotype.

Lago Tupé beach, lower Rio Negro tributary, Manaus, Amazonas, Brazil (003°02'42"S, 060°15'10"W), ♂, col. FL Carvalho and EA Souza-Carvalho, 27 January 2012 (CCDB 4865).

#### Paratypes.

same data as holotype, 28♂ 8♀ 17♀ov (CCDB 4866); same data as holotype, 10♂ 3♀ 3♀ov (INPA 2016); same data as holotype, 1♂ 1♀ 1♀ov (OUMNH-ZC 2013-08-001).

#### Other material.

***Brazil*.** *Amazonas*. Parque Nacional de Anavilhanas, lake near the rio Apuaí’s mouth, 1♂ 1♀, col. J Zuanon, 20 August 2005 (INPA 1432); Manaus, Rio Negro basin, Igarapé do Camarão, 20♀ov, col. O Odinetz-Collart et al., 28 February 1989 (CCDB 4726); Rio Negro basin, igarapé Alagadiço, 20♂, col. O Odinetz-Collart et al., 17 January 1989 (CCDB 4727); São Gabriel da Cachoeira, igarapé Barixia, right bank of the Rio Negro, 4♀, col. J Cunha et al., 14 December 2005 (MZUSP 16907); Santa Izabel do Rio Negro, 2♀, 24 October 1972 (MZUSP 13645); rio Uatumã, near the igarapé do Miriti’s mouth, 1♂ 3♀, col. C Magalhães, 12 July 1985 (INPA 173).

#### Diagnosis.

Mandibular palp absent. Rostrum slender, slightly curved upward at the distal half, overreaching the scaphocerite; dorsal margin with 6 to 10 teeth; ventral margin with 2 to 5 teeth, usually 3 or 4. Projection of the anterolateral margin of the first antennular segment reaching the dorsal distal margin of the second segment; anterolateral spine of the first antennular segment not overreaching the first third of the projection of the anterolateral margin. Appendix masculina up to 1.1 the length of the appendix interna, measured from their junction. Telson carrying 2 plumose setae between the inner distal stout setae; inner distal stout setae overreaching the distal tip of the telson.

#### Description.

Carapace glabrous. Sub-orbital lobe and pterygostomial angle rounded. Branchiostegal suture located approximately with a half of the distance between the antennal and branchiostegal tooth. Branchiostegal tooth almost as strong as the antennal, placed behind the anterior margin of the carapace.

Rostrum slender, slightly curved upward at the distal half, overreaching the scaphocerite (Fig. 7a); dorsal margin with 6 to 10 teeth, 5 to 8 of them placed in the proximal 2/3, 1 or 2 dorsal teeth located behind the orbit, 1 or 2 subapical teeth; ventral margin with 2 to 5 teeth, usually 3 or 4, all of them placed in the distal half. Single row of setae present on the proximal ventral portion up to the second tooth; double continuous and uniformly spaced row of setae on the distal half.

Eye well developed with pigmented cornea (Fig. 7b); cornea slightly wider and smaller than the eyestalk; ocellus present on dorsal side.

Antennular peduncle not reaching the distal margin of the scaphocerite; first antenular segment with outer margin slightly convex and projection of the anterolateral margin rounded, reaching the dorsal distal margin of the second segment (Fig. 5c); anterolateral spine of the first antennular segment not overreaching the first third of the projection of the anterolateral margin; second segment as broad as and shorter than the third segment; inner ventromesial tooth present; upper antennular flagellum fused for about 1/5 of its length (4–6 segments fused, 14–16 free); free portion with two rows of two or three aesthetascs on each segment. Stylocerite short, not reaching the middle of the first antennular segment. Béc ocellaire with anterior margin concave, pronounced upwardly and bearing a pigmented spot dorsally.

Scaphocerite slender (Fig. 7c), laminar, 3.3 times as long as broad; outer margin slightly concave, terminating in a tooth, not overreaching the lamella; basal segment of antenna with strong lateral tooth. Flagellum of the antenna more than five times the length of the body.

Mandibular palp absent; incisor process with three teeth on both sides (Fig. 7d). Upper lacina of the maxillula just reaching the length of the inner lacina (Fig. 7e). First maxilliped with lobes of the epipod fused (Fig. 7h–i); anterior lobe elongated and almost triangular, with lateral border slightly convex; junction between the endites roundly curved. Epipod of the third maxilliped with anterior margin forming an angle of about 45° (Fig. 7j).

Thoracic sternal armature sexually similar. First thoracic sternite with an acute tooth and a conspicuous transverse ridge; second without tooth, bearing a triangular transverse ridge. Third to fifth without tooth and ridge incomplete.

First pereiopod slender (Fig. 7l), reaching the tip of scaphocerite with the fingers; ischium 1.8 times the length of basis; merus 1.7 times the length of ischium; carpus 1.3 times the length of merus; chela slightly less than 0.5 the length of carpus; fingers as long as palm (Fig. 7m).

Second pereiopod slender (Fig. 7k), overreaching the scaphocerite with about a half of the inflated distal part of the carpus; ischium about 4.5 times the length of basis; merus 0.8 the length of ischium; carpus 1.8 times the length of merus; chela about 0.6 times the length of carpus; fingers about 0.7 the length of palm (Fig. 7n).

Third pereiopod (Fig. 8a) slender, reaching the tip of the scaphocerite; ischium 1.8 times the length of basis; merus 1.9 times the length of ischium; carpus about 0.5 times the length of merus; propodus 1.6 times the length of carpus, ventral margin armed with 5–8 cuspidate setae; dactylus simple, about 0.3 times the length of the propodus.

Fourth pereiopod slender (Fig. 8b), overreaching the scaphocerite with all length of the dactylus; ischium 1.7 times the length of basis; merus 2.4 times the length of ischium; carpus about 0.5 length of merus; propodus 1.7 times the length of carpus, ventral margin armed with 7-13 cuspidate setae; dactylus simple, about 0.3 the length of propodus.

Fifth pereiopod slender (Fig. 8c–d), overreaching the scaphocerite with the end of the propodus; ischium 1.7 times the length of basis; merus 2.3 times the length of ischium; carpus about 0.6 length of merus; propodus 2.1 times the length of carpus; grooming brush comprises about 10 rows of setae on the distal third of the propodus; dactylus simple, about 0.2 the length of propodus.

First pleopod without appendix interna and sexually dimorphic in proportions; males with endopod 0.5 the length of exopod (Fig. 8e); females with endopod approximately 0.3 length of exopod (Fig. 8f). Second to fifth pleopods similar, with the endopod reaching about 0.8 the length of exopod and bearing an appendix interna (Fig. 8g). Appendix masculina up to 1.1 times the length of the appendix interna, measured from their junction (Fig. 8h).

Abdominal sternal armature sexually dimorphic; males with first and second sternites bearing median process; second more acute and bigger than the first process; females with median process less develop than males.

Abdominal pleura furnished with plumose setae on ventral margin; fifth pleuron elongated and disto-ventrally rounded (Fig. 8i), with dorsal posterior border concave; sixth segment 1.63 times the length of the fifth; posterolateral margin with small tooth and keel disto-ventrally. Anal fig unarmed (Fig. 8j).

Telson as long as sixth pleonite; dorsal surface with two pairs of cuspidate setae (Fig. 8k); proximal dorsal tuft of setae reduced to one or two simple setae; marginal setae absent; posterior margin ending abruptly in a triangular tip, not overreaching the inner stout setae (Fig. 8l); 1 pair of plumose setae and 2 pairs of stout setae, inner pair of stout setae about 2.3 times the length of the outer pair.

Uropods overreaching the telson by 0.3 of the length of exopod; exopod 1.25 times the length of endopod; mobile distolateral setae of exopod weak, reaching about the middle of the fixed tooth (Fig. 8m).

#### Geographic distribution.

Brazil (Amazonas, Pará?), Venezuela? (Apure).

#### Ecological features.

Usually associated with riparian vegetation, leaf litter and similar microhabitats in lakes, streams and rivers of black or clear water river systems, in areas with low flow.

#### Etymology.

**Figure 9.**
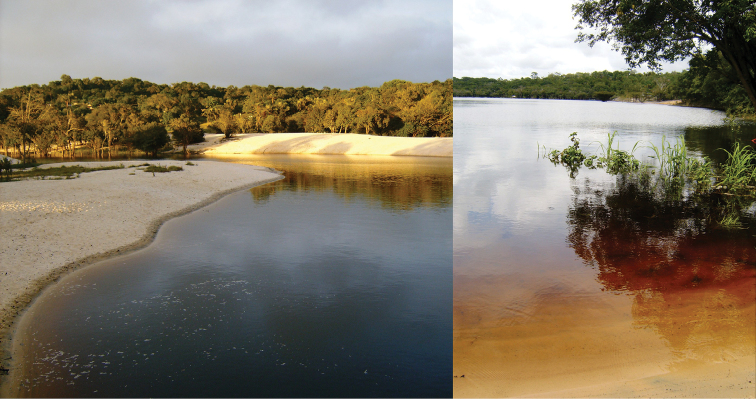
Type locality of *Palaemon yuna* sp. n. Lago Tupé beach, lower Rio Negro tributary, Manaus, Amazonas, Brazil (003°02'42"S, 060°15'10"W).

The specific epithet is derived from the Tupi, the general language of the Brazilian indigenous people: *y* = water, river + *úna* = black, alluding to the environment where the species was first found (Fig. 9).

### Key for the species of the *Palaemon carteri* / *Palaemon ivonicus* / *Palaemon yuna* sp. n. group

**Table:** 

1	Projection of the anterolateral margin of the first antennular segment not overreaching the middle of the second segment	**other South American Palaemoninae**
–	Projection of the anterolateral margin of the first antennular segment overreaching the middle of the second segment	**2**
2	Mandibular palp present	**other South American Palaemoninae**
–	Mandibular palp absent	**3**
3	Projection of the anterolateral margin of the first antennular segment not reaching, rarely almost reaching, the dorsal distal margin of the second segment; anterolateral spine of the first antennular segment usually reaching the middle of the projection of the anterolateral margin (Fig. 5a); rostrum with 3 to 7 ventral teeth (usually 4 or more)	***Palaemon carteri***
–	Projection of the anterolateral margin of the first antennular segment reaching or overreaching the dorsal distal margin of the second segment; anterolateral spine of the first antennular segment not reaching the middle of the projection of the anterolateral margin (Fig. 5b–c); rostrum with 1 to 5 ventral teeth (usually 4 or fewer)	**4**
4	Rostrum high, straight or slightly curved upward, not overreaching the scaphocerite; rostrum with 1 to 4 ventral teeth (usually 3 or fewer) (Fig. 6b). Anterolateral spine of the first antennular segment generally reaching the first third of more of the projection of the anterolateral margin (Fig. 5b)	***Palaemon ivonicus***
–	Rostrum slender, slightly curved upward, overreaching the scaphocerite; rostrum with 2 to 5 ventral teeth (usually 3 or 4) (Fig. 6c). Anterolateral spine of the first antennular segment generally not reaching the first third of the projection of the anterolateral margin (Fig. 5c)	***Palaemon yuna* sp. n.**

## Discussion

Both molecular and morphological data support the validity of *Palaemon ivonicus*, refuting the hypothesis that this species is a junior synonym of *Palaemon carteri*. The two species are allocated in two sisters Amazonian lineages, with great genetic divergence and morphological support. Additionally, a third species closely related to *Palaemon ivonicus* is described.

The projection of the anterolateral margin and anterolateral spine of the first antennular segment seems to be an important character in this group, although we have found some specimens with a state of character close to an intermediate form, making difficult a clear distinction between these lineages. Additionally, there is an ontogenetic variation, which needs to be considered in the analyses. Some large specimens of the “carteri” lineage have the antennular projection almost reaching the dorsal margin of the second segment. On the other hand, some small specimens of the “ivonicus/yuna” lineage also have the projection almost reaching the dorsal margin of the second segment. However, even considering those limitations, the antennular character was the most constant one to distinguish the “carteri” and “ivonicus/yuna” lineages. Therefore, we suggest that this character must be evaluated in further morphological analyses within the *Palaemon* genus.

The wide intraspecific morphological variability and interspecific similarity between *Palaemon carteri* and *Palaemon ivonicus* as well as the presumptive synonymy have been reported by previous studies since the 1970s (Gomes-Corrêa 1977, Odinetz-Collart and Enriconi 1993, García-Dávila and Magalhães 2003, García-Dávila et al. 2005). Despite of that similarity posteriorly reported, Holthuis (1950a, 1952) did not make a direct comparison of *Palaemon carteri* and *Palaemon ivonicus* in order to clearly differentiate them. Furthermore, the type series of *Palaemon ivonicus* is composed by only two specimens, making it impossible to evaluate the morphological variability spectrum within this species. Therefore, it is not surprising that misidentification may occur, which may have contributed to the hypothesis of synonymy.

The overlapping of several morphological characters demonstrated by Odinetz-Collart and Enriconi (1993) and García-Dávila et al. (2005) probably is due to the fact that all or most of the specimens examined by these authors were from the “ivonicus/yuna” lineage. These studies used the traditional rostral characters to identify some specimens from the central Amazon basin as *Palaemon carteri*. We were unable to find any sample of *Palaemon carteri* in the central and western Amazon basin, since specimens from these regions are more likely to be assigned to the “ivonicus/yuna” lineage. Therefore, the rostral similarity between *Palaemon carteri* and *Palaemon yuna* sp. n. probably has led many authors to use the name *Palaemon carteri*, which was the name available at the moment that better fitted the rostral characteristics of some populations from the central Amazon basin (see synonymic list of *Palaemon yuna* sp. n.).

**Figure 10.**
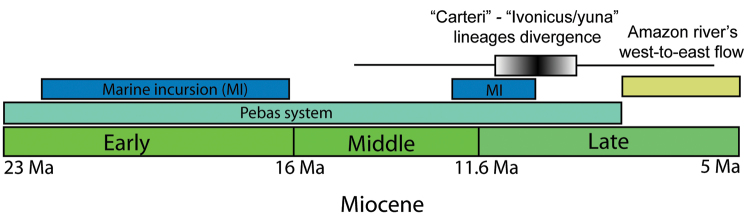
Historical context for the proposed divergence time between the “carteri” and “ivonicus/yuna” lineages.

**Figure 11.**
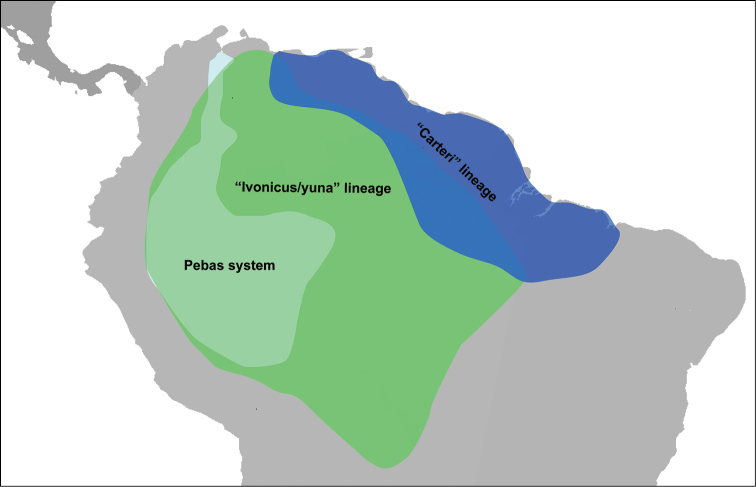
Putative current distribution of the “carteri” and “ivonicus/yuna” lineages. [Pebas system during the Late Miocene (~11.8 to 10 Ma) according to Lundberg et al. 1998.]

The wide genetic dissimilarity between the “carteri” and “ivonicus/yuna” lineages shows that they have no recent divergence, as one could expect based only on their morphological similarity. The approximately 10 million years of divergence estimated for these lineages, based on the 16S rRNA gene, may be associated with marine incursion as well as colonization of different environments in western Amazonia during the Middle Miocene (~16 to 11.6 Ma) and Late Miocene (~11.6 to 5.3 Ma) (Fig. 10). From the Early Miocene until the early Late Miocene (~23 to 9 Ma), the western Amazonia region was mostly submerged and transformed into a continually shifting mosaic of lakes, wetlands and river belts (Wesselingh 2006), which are similar to the environments where specimens of the “ivonicus/yuna” lineage are currently found. This continually shifting mosaic, the Pebas system, had contact with the Caribbean Sea and underwent several marine incursions during the Middle and Late Miocene (Hoorn 1993, Wesselingh 2006). Particularly in the period between 11.8 and 10 Ma, close to our estimate for the time of divergence between the “carteri” and “ivonicus/yuna” lineages, there is evidence of an extensive marine transgression into the low-lying basins of South America (Lundberg et al. 1998). These marine incursions, which established brackish-water conditions in the late Middle Miocene and early Late Miocene (Hoorn 1993, Lundberg et al. 1998), might have isolated freshwater lineages of the Pebas system from others distributed in northern South America outside this wetland system, where the “carteri” lineage is currently found (Fig. 11). Similar biogeographical patterns and speciation events associated with the Pebas system and marine incursions in the Middle and Late Miocene have been reported for several groups in the Amazon region (Hoorn 1993, Lovejoy et al. 1998, 2006, Lundberg et al. 1998, Wesselingh 2006, Santos et al. 2009, Cooke et al. 2012a,b). Therefore, the proposed current parapatric distribution of the “carteri” and “ivonicus/yuna” lineages probably developed after the establishment of the modern west-to-east course of the Amazon River, which may have initiated approximately 8 Ma (Lundberg et al. 1998).

The specimens from the Negro River (*Palaemon yuna* sp. n.) have considerable genetic divergence from the specimens collected in the Solimões-Amazon and Xingu rivers, being allocated outside the *Palaemon ivonicus* *sensu stricto* clade. The specimens from the Negro River also show some differences in the rostrum, in the antennular characters and in the appendix masculina compared to specimens from the type locality of *Palaemon ivonicus* as well as other basins (Figs 5–6). Additionally, a morphometric study with specimens from the Negro River revealed that they form a morphometrically distinct group from populations that inhabit white water, although some overlapping was found (García-Dávila et al. 2005). Studies with fishes reported a similar pattern, providing evidence for the effect of divergent natural selection associated with the difference in water colour between the Negro River and Solimões-Amazon River (Cooke et al. 2012a,b). Genetic divergence between hydrologically different, but interconnected, environments has been reported for other palaemonid species without a conspicuous and consistent morphological differentiation (Carvalho et al. 2013). However, the characters described above allow the morphological distinction between *Palaemon ivonicus* and *Palaemon yuna* sp. n. Therefore, regarding the genetic, morphological and ecological differentiation, there is sufficient evidence to justify the proposal of a new species for the populations from the Negro River basin. The actual distributional range of *Palaemon yuna* sp. n. may be wider than indicated by the material available for this study. Specimens from the lower Tapajós River basin might be co-specific with the specimens from the Negro River basin, as suggested by the results of a molecular study conducted by García-Dávila (2002) in an unpublished academic thesis, using the mitochondrial cytochrome c oxidase subunit I gene (COI).

Specimens from the upper Orinoco River basin have antennular characters similar to those of *Palaemon yuna* sp. n. Nevertheless, some specimens fail to have a curved upward rostrum overreaching the scaphocerite. The connection between the Negro and Orinoco river basins through the Casiquiare River opens the possibility of the existence of a conspecific group occurring in the Negro and Orinoco rivers. A similar biogeographical pattern has been reported for fish species, using molecular data (Willis et al. 2010). However, despite logistic difficulties, a more thorough genetic and morphological sampling along the Negro River and the upper Orinoco River basins should be carried out in order to verify the phylogenetic relationships among the populations from the upper Orinoco and other populations of the “ivonicus/yuna” lineage as well as its taxonomic status. Similarly, the records of *Palaemon ivonicus* from the Orinoco River basin (López and Pereira 1996, 1998, Pereira et al. 2010a, b) should be verified, since morphologically (judged by the illustration provided by López and Pereira 1996: 55, fig. 9) and zoogeographically these specimens probably do not belong to this taxon.

The occurrence of *Palaemon ivonicus* in the Paraguay/lower Paraná River basin is an issue that needs further analyses in order to verify the phylogenetic relationships of these populations. As discussed by Magalhães et al. (2005), the decapod fauna of the Amazon and the Paraguay-Paraná river lowlands has several common elements whose current distributions may be result of dispersal across the paleobasins of these systems during Tertiary and Quaternary as some geological events changed their boundaries promoting different sequences of capture of headwater (Lundberg et al. 1998). Even in recent times, sporadic or seasonal contact between the Amazon and the Paraguay-Paraná fluvial nets can occur, as some landscape features along their boundary favor transfluences, headwater captures, floods and spillouts to one or other side (Iriondo and Paira 2007).

An additional record of *Palaemon ivonicus* from the São Francisco River basin, state of Minas Gerais, Brazil (Melo 2003), cannot be confirmed as it was not documented by voucher material, and we were unable to find any samples from this basin in the collections visited by us. In addition, we could not find any individual of this species in field collections made in that state. Moreover, the geological history of the São Francisco River basin does not show any evidence of a connection with the Amazonian basin during the Neogene or Quaternary period, which makes unlikely a natural occurrence of *Palaemon ivonicus* in this basin. The report of *Palaemon carteri* for Mexico (Felder et al. 2009) seems to be an error, since the notes associated to the species do not match with the distribution and habitat of the species, and no other studies have reported this species for the Gulf of Mexico.

The fact that our target group seems to be closer related to three neotropical species of *Palaemon* (*Palaemon gracilis*, *Palaemon hancocki* and *Palaemon pandaliformis*) than to other species of the genus require further studies. Ashelby et al. (2012) recovered *Palaemon gracilis* and *Palaemon pandaliformis* outside the clade that comprised the majority of the species of *Palaemon*. Therefore, *Palaemon carteri*, *Palaemon ivonicus* and *Palaemon yuna* might also not have a close relationship with *Palaemon* *sensu stricto*. However, as this was not the goal of the present study, a broader taxonomic sampling of the subfamily using both mitochondrial and nuclear genes is needed to address properly these generic-level questions.

Our study is part of a project aiming to investigate the American species of *Palaemon*, and this is the first one which uses a multidisciplinary approach aiming to clarify this taxonomic issue. Our data clearly show that there are at least two morphologically and genetically distinct lineages, which might have diverged ~10 Ma. A multilocus approach is needed to provide more molecular support for this estimated divergence time. The possibility of hybridization cannot be rejected and must be deeply investigated in further studies. Moreover, the morphological variability found in some populations still assigned to *Palaemon ivonicus* as well as the molecular variability found within the “carteri” lineage need to be further investigated to verify whether there are other morphologically similar species not yet described.

## Supplementary Material

XML Treatment for Palaemon carteri

XML Treatment for Palaemon ivonicus

XML Treatment for Palaemon yuna
